# The Relationship Between Personal Recovery and Experienced Loneliness, Discrimination or Neighborhood Unsafety in Patients With a Schizophrenia Spectrum Disorder

**DOI:** 10.1177/00207640261438835

**Published:** 2026-04-09

**Authors:** Auke Jelsma, Marieke van der Pluijm, Menno Segeren, Astrid Vellinga, Henriëtte Heering, Annet van Bergen, Martijn Kikkert, Mariken de Koning, Lieuwe de Haan

**Affiliations:** 1 Department of Psychiatry, Amsterdam UMC Location University of Amsterdam, The Netherlands; 2 Arkin Institute for Mental Health, Amsterdam, The Netherlands; 3 Department of Healthy Living, Public Health Service Amsterdam, The Netherlands; 4 Department of Research & Innovation, GGZ InGeest, Amsterdam, The Netherlands

**Keywords:** personal recovery, loneliness, discrimination, unsafety, victimization, schizophrenia spectrum disorders, psychosis

## Abstract

**Background:**

Personal recovery for patients with a schizophrenia spectrum disorder (SSD) is a process of finding meaning in life after being confronted with psychiatric symptoms. Currently, not much is known about the impact of patients’ living environments and social interactions. The relationships between personal recovery and experiences of loneliness, discrimination and unsafety remain scarcely explored for patients with SSD. Understanding these relationships may contribute to developing recovery-supportive treatment strategies and improve collaboration between medical and social services. The current study aimed to examine the relationships between personal recovery and experiences of loneliness, discrimination, or neighborhood unsafety in patients with SSD receiving outpatient treatment.

**Methods:**

Hierarchical regression analyses were performed in 204 patients to assess the explained variance in personal recovery by loneliness, discrimination and neighborhood unsafety, after adjusting for a priori defined covariates. The potential interaction effects of symptom severity with the three predictors were also investigated.

**Results:**

Loneliness explained 9.6% additional variance in personal recovery (β = −.34, *p* < .001). Subjective unsafety contributed modestly by explaining 2.8% (β = −.17, *p* = .012) of additional variance in personal recovery, while severity of unsafety did not contribute. Discrimination did not significantly explain additional variance in personal recovery. No significant interaction effects between symptom severity and the three predictors were observed in the associations.

**Conclusion:**

Current findings demonstrate a negative association between loneliness and personal recovery in patients with SSD, while subjective unsafety was modestly negatively associated with personal recovery. Addressing loneliness deserves special attention in mental healthcare and social services, highlighting the need for close collaboration between these domains.

## Introduction

Different definitions of recovery are used for patients with schizophrenia spectrum disorders (SSD). Clinical recovery is the conventional perspective on recovery, typically defined as the reduction of psychotic symptoms as assessed by healthcare practitioners (Andreasen et al., 2005; Asbo et al., 2022). Social or functional recovery entails recovering of patients’ level of daily functioning, including social or family relationships and their working and living situations (Castelein et al., 2021). In recent years, more attention has been directed toward personal recovery for patients with SSD. The concept of personal recovery has emerged from the patient movement and is best understood as a personal process or journey in which meaningfulness in life is found despite psychiatric symptoms (Anthony, 1993; Slade et al., 2008; van Weeghel et al., 2019). Connectedness, hope, identity, meaningfulness and empowerment (forming the acronym CHIME together) have been proposed as elements for a conceptual framework of personal recovery, based on a synthesis of people’s experiences of recovery in mental illness (Leamy et al., 2011).

Given that negative experiences in patients’ living environments and social interactions may hinder personal recovery, understanding their impact could be relevant for improving care for community-treated patients. Prior studies reported that patients with SSD often experience reduced social support and more loneliness than the general population (Badcock et al., 2015; Gayer-Anderson & Morgan, 2013; V. A. Morgan et al., 2012; Norman et al., 2012). To our knowledge, only one study assessed the relationship between loneliness and personal recovery in this group, observing that higher loneliness was associated with lower personal recovery (Roe et al., 2011).

Research indicates that patients with SSD are more vulnerable to discrimination, which has been proposed to contribute to the risk of developing psychosis (Lazaridou et al., 2023; Misra et al., 2021; Pearce et al., 2019). In patients with severe mental illness, a negative association was observed between perceived discrimination and personal recovery (Mak et al., 2017). A similar negative association was observed in patients with first-episode psychosis (Laxmi et al., 2023). Additionally, in patients with severe mental illness higher perceived discrimination was associated with decreased access to social networks (Webber et al., 2014).

Research has also shown that patients with SSD are more susceptible to criminal victimization than the general population (Bengtsson-Tops & Ehliasson, 2012; Dean et al., 2007). One study observed a correlation between violent victimization and both higher psychotic symptom severity and poorer social functioning in patients with psychosis (Chapple et al., 2004). Less is known about how patients’ experiences of neighborhood unsafety relate to recovery. Studies involving patients with severe mental illness reported negative associations between perceptions of neighborhood safety and psychiatric distress, and positive associations between better neighborhood perception and both well-being and self-reported recovery (Kloos & Townley, 2011; Wright & Kloos, 2007). Currently, to the best of our knowledge, no study has assessed the relationship between subjective neighborhood unsafety and personal recovery in SSD patients.

Currently, little is known about how perceptions of their living environments and social interactions relate to personal recovery for patients with SSD. A deeper understanding of the relationships between experiences of loneliness, discrimination and neighborhood unsafety on the one hand, and the level of personal recovery on the other hand, could provide valuable insights into patients’ personal recovery process. Such insights could elucidate potential psychosocial targets for intervention. They may also highlight the necessity of collaboration between mental healthcare and social services, enabling a more integrated care approach.

The current study aimed to examine the relationships between personal recovery and experiences of loneliness, discrimination, and neighborhood unsafety in community-treated SSD patients. A secondary aim was to explore the potential interactional effect between symptom severity and these experiences on personal recovery, prompted by prior literature indicating positive correlations between these experiences and symptom severity (Meijwaard et al., 2015; Michalska da Rocha et al., 2018; Pearce et al., 2019; Wang et al., 2020), as well as the established negative association between symptom severity and personal recovery (Castelein et al., 2025; Leendertse et al., 2021; Van Eck et al., 2018), potentially implying a negative interaction effect.

## Method

### Study Sample and Design

For the current cross-sectional study data were used from the “Deinstitutionalization Amsterdam” research cohort, a group of 650 adults with chronic severe mental illness receiving outpatient treatment from the two largest mental health care providers in Amsterdam. Participants were sampled from the city’s quarters distributed across residential areas, based on their postal codes, to ensure a sample representative of the city, its districts and quarters. All participants were aged between 18 and 65 years old and met criteria for a severe mental illness according to the Dutch definition, consisting of having a psychiatric disorder with ongoing symptoms for a prolonged period of time (at least several years), associated with serious social impairments and requiring coordinated, integrated care (Delespaul & de consensusgroep, 2013). Exclusion criteria were intellectual disability, dementia, staying in long-term clinical locations and lack of proficiency in Dutch or English. Participants were selected after their healthcare practitioners assessed their eligibility and where then approached by a research assistant. The full cohort and study methods have been described in detail elsewhere (Segeren et al., 2022).

The study was granted exemption from ethical approval by the Medical Ethical Review Committee of Amsterdam UMC (code: W16_276 # 16.324). All participants provided written informed consent and received 15 euros for participation. Structured interviews were carried out by research assistants affiliated with the mental health care institutions using validated instruments. Research assistants were master’s level psychology students, who received specific training in psychiatric symptom assessment with the BPRS-E. Data were collected between December 2017 and February 2020. For the current study, only participants with a schizophrenia spectrum or other psychotic disorder (SSD) according to DSM-5 criteria (American Psychiatric Association, 2013) were included. Participants with other diagnoses (379), no formal outpatient treatment status (11) or incomplete data (56) were excluded, leading to a final sample of 204 participants; 61.3% had a diagnosis of schizophrenia.

### Measurements

#### Personal Recovery

The Questionnaire of Personal Recovery (QPR) was used to measure the level of personal recovery. The QPR is a self-report instrument with good psychometric properties (Felix et al., 2024; Law et al., 2014). It encompasses 22 items scored on a 5-point scale ranging from 1 (*strongly disagree*) to 5 (*strongly agree*), with higher scores indicating higher levels of self-reported personal recovery. The QPR consists of two subscales, corresponding to an intrapersonal (17 items: 1–13, 18, 19, 21, 22; e.g., “I feel that my life has a purpose”) and an interpersonal (5 items: 14–17, 20; e.g., “meeting people who have had similar experiences makes me feel better”) domain (Neil et al., 2009). Sum scores were calculated using all items for total personal recovery scores (range 22–110) and for each subdomain (See Supplemental Material ST1 for the full questionnaire).

#### Loneliness

The short version of the De Jong-Gierveld Loneliness Scale was used to measure the level of experienced loneliness. It has shown reliability and validity in measuring loneliness (de Jong Gierveld & van Tilburg, 2008; van Tilburg & de Jong Gierveld, 1999) and has been used in SSD patients before (Chrostek et al., 2016). Participants rated six statements (e.g., “There are many people that I can count on completely”), each scored on a 5-point Likert scale, ranging from 1 (*strongly disagree*) to 5 (*strongly agree*). Following previous research (Rodriguez-Blazquez et al., 2021), a sum score was used after negatively recoding (to ensure all items aligned in the same direction) and dichotomizing (⩾3 indicating loneliness, as this score reflects the presence of loneliness for the specific item), with higher scores indicating more experienced loneliness (range 0–6).

#### Discrimination

The Everyday Discrimination Scale (EDS) was used to assess subjective discrimination. Originally developed in the United States to measure everyday discrimination experiences in ethnic minority groups, it shows good psychometric properties in the general population (Krieger et al., 2005; Williams & Mohammed, 2009). Participants rated 9 statements, each scored on a 4-point Likert scale, ranging from 1 (*often*) to 4 (*never*). Higher scores, after negatively recoding items, indicated more frequent experiences of discrimination. A sum score was used (range 9–36).

#### Subjective Unsafety and Victimization

Subjective neighborhood unsafety was assessed with the Dutch Integral Safety Monitor (ISM; Integrale Veiligheidsmonitor in Dutch), a self-report tool commissioned by the Ministry of Safety and Justice to evaluate neighborhood livability, subjective safety and victimization in all Dutch municipalities periodically. Although no formal psychometric evaluation exists, the ISM is regarded as reliable tool for victimization data in the Netherlands (Centraal Bureau voor de Statistiek, 2019; Segeren et al., 2022) and has been used in studies with patients with severe mental illness before (de Mooij et al., 2015; Meijwaard et al., 2015).

Two variables were used to measure neighborhood unsafety. A binary variable based on the question “Do you sometimes feel unsafe in your own neighborhood?” was employed to assess if neighborhood unsafety was occasionally experienced. The severity of subjective unsafety was measured with five follow-up questions in the subgroup who reported neighborhood unsafety (see Supplemental Material ST2), scored on a 3-point Likert scale, ranging from 1 (*rarely* or *never*) to 3 (*often*). A sum score was calculated (range 5–15).

The ISM was also used to assess victimization frequency. Participants reported whether or not they had been a victim of 15 specific victimization categories in the past 12 months (see Supplemental Material ST3). A binary variable was constructed indicating whether any victimization had occurred.

#### Covariates

Age, sex, educational level and country of birth were selected as covariates based on known associations with personal recovery (de Winter et al., 2024) or recovery outcomes (Griffiths et al., 2023; C. Morgan et al., 2019) in SSD patients. Cannabis (Bruins et al., 2021), alcohol (Carra et al., 2016) and nicotine use (Vermeulen et al., 2019) were selected considering established associations with recovery. Hazardous alcohol use was measured with the Alcohol Use Disorders Identification Test (AUDIT), using gender-specific cutoffs (⩾8 for males and ⩾6 for females; Cetty et al., 2019; van der Heijden et al., 2024) and recoded into a binary variable. Cannabis and nicotine were measured with the Measurements in the Addictions for Triage and Evaluation (MATE) and dichotomized based on use in the past 30 days. Overall symptom severity, based on known associations with personal recovery (Van Eck et al., 2018), was measured with the Brief Psychiatric Rating Scale (BPRS-E) on a 7-point Likert scale (*not present* to *very severe*), including 14 interview-rated and 10 observer-rated items (Thomas et al., 2004). A sum score of the four symptom subscales of the BPRS-E (positive, negative, affective and excitement/disorganization symptoms) based on prior literature (Ruggeri et al., 2005) was used with higher scores indicating higher total symptom severity (range 24–168).

#### Analysis

Data were analyzed using IBM SPSS Statistics 28. Participants were included if they had data for at least 80% of items of relevant variables, assuming that missingness was not completely at random. Prior to analyses, regression assumptions were checked. Histograms and scatter plots were visually inspected for normality of distribution of residuals, outliers and homoscedasticity. Multicollinearity was examined using Variance Inflation Factor (VIF) and tolerance. To address missing covariate data and maintain statistical power, sensitivity analyses were conducted using multiple (5 datasets) imputations (solely for covariates).

To examine the unique contribution of loneliness, subjective discrimination and subjective neighborhood unsafety to personal recovery while controlling for covariates, separate multiple hierarchical regression analyses were conducted. Sociodemographic covariates were entered in block 1, total symptom severity and substance use in block 2 and the independent variable in block 3. Interaction effects between symptom severity and the independent variable on personal recovery were examined by adding the interaction term in block 4, using centered variables to minimize multicollinearity. Bonferroni multiple testing correction was applied based on three different models (*p* = .05 / 3 = .017). An exploratory analysis was conducted to examine specific interaction effects between loneliness and four symptom subscales on personal recovery, as prior literature suggests different relationships between loneliness and various symptom categories (Winkler et al., 2024). Pairwise exclusion, excluding participants only when data for relevant variable pairs were missing, was used to minimize data loss.

Analyses for the three predictors were also conducted within the subsample of patients diagnosed with schizophrenia, given its diagnostic specificity. A post-hoc model was conducted in the subgroup reporting neighborhood unsafety to assess whether subjective unsafety severity explained additional variance in personal recovery. Additionally, an exploratory regression analysis was conducted to triangulate findings on personal recovery and subjective unsafety, examining whether victimization explained additional variance in personal recovery.

Two exploratory regression analyses were conducted with the interpersonal and intrapersonal recovery subscales as dependent variable, to examine whether the association between loneliness and personal recovery would solely depend on conceptual overlap between loneliness and the interpersonal subscale.

## Results

### Baseline Characteristics and Sensitivity Analyses

Sociodemographic variables, diagnoses and an overview of outcome scores are displayed in Table 1. Imputed analyses for missing covariates for all models showed no significant deviations from original analyses, suggesting missing covariates were at random and did not significantly impact the results (Supplemental Material ST4).

**Table 1.** Sociodemographic and Clinical Characteristics for the Total Sample (*n* = 204).

Characteristic / Variable	*n* (%) / Mean (*SD*)*
Age (years)	*n =* 204 47.94 (9.52)
Sex, *n* (%)	*n =* 202
Male	133 (65.2)
Female	69 (33.8)
Highest level of education attained,^a^ *n* (%)	*n =* 203
No education	6 (3.0)
Primary education	121 (59.6)
Secondary education	56 (27.6)
Higher education	20 (9.9)
Birth country, *n* (%)	*n =* 202
South-America, Africa, or Asia^b^	85 (41.7)
Europe, North-America or Oceania^c^	117 (57.4)
Classification according to DSM IV criteria, *n* (%)	*n =* 204
Schizophrenia	125 (61.3)
Schizophreniform disorder	1 (0.5)
Schizoaffective disorder	39 (19.1)
Delusional disorder	3 (1.5)
Brief psychotic disorder	35 (17.2)
Psychotic disorder due to medical condition	1 (0.5)
Living situation, *n* (%)	*n =* 204
Independent and single	128 (62.7)
Independent and with partner	20 (9.8)
With parents or family or friends	17 (8.3)
Sheltered housing in the community	29 (14.2)
Social pension (24-hr facility)	6 (2.9)
Nursing home	3 (1.5)
Other**	1 (0.5)
Personal recovery score (QPR; range 22–110)	*n =* 204 81.58 (10.51)
* Intrapersonal scale* (range 17–85)	*n =* 204 64.070 (9.12)
* Interpersonal scale* (range 5–25)***	*n =* 193 17.92 (2.59)
Loneliness (range 0–6)	*n =* 203 2.61 (1.83)
Subjective discrimination (range 9–36)	*n =* 202 16.31 (6.36)
Reporting feeling unsafe in the neighborhood occasionally, *n* (%)	*n =* 203 65 (31.9)
Subjective unsafety severity (range 5–15)	*n =* 57^d^ 8.44 (2.50)
Victimization (last 12 months), *n* (%)	*n =* 204 73 (35.8)
BPRS-E total symptom severity score (range 7–168)	*n =* 202 37.28 (10.078)
Positive symptoms (range 5–35)	*n =* 202 8.78 (4.59)
Negative symptoms (range 7–49)	*n =* 202 10.094 (2.99)
Affective symptoms (range 6–42)	*n =* 201 10.56 (4.19)
Excitement/Disorganization symptoms (range 9–63)	*n =* 202 12.23 (3.39)
Cannabis use (last 30 days), *n* (%)	*n =* 199 42 (20.6)
Nicotine use (last 30 days), *n* (%)	*n =* 202 109 (53.4)
Hazardous alcohol (last year),^e^ *n* (%)	*n =* 201 42 (20.6)

a Based on the Dutch educational system.

b Patients born in Morocco (10), Turkey (4), Surinam (44), the Netherlands Antilles (11), or another non-specified country from South-America, Africa, or Asia (23).

c Patients born in the Netherlands (112) or another non-specified country from Europe, North-America or Oceania (9).

d Within the group reporting feeling unsafe sometimes: 8 participants of the 65 participants reporting feeling unsafe had insufficient data for this variable.

e AUDIT score ⩾8 for males, 6⩾ for females.

* For categorical variables, number (*n*) and percentages (%) of participants are reported for the specific variable. For ordinal/continuous variables, means and standard deviations (*SD*) are reported with two decimal places for the specific variable. **For instance, staying in detention, at a residential hotel, family replacement home, with ex-partner, boarding school or asylum seekers’ center. ***Due to a smaller number of items in the interpersonal subscale, participants were more likely to have missing data at random, given that the required 80% of items per variable was easier to fail. This resulted in more dropout of participants for the interpersonal subscale compared to the intrapersonal subscale.

### Multiple Hierarchical Regression Analysis Results

#### Loneliness

Visual inspection showed a normal distribution of loneliness (*n* = 203). One hundred ninety-six participants were included in the model, as 7 were excluded pairwise due to missing variable pairs. Sociodemographic covariates (block 1) did not explain variance in personal recovery. Adding symptom severity and substance use (block 2) explained 12.6% additional variance in personal recovery (*R*^2^ change = .126, *p* < .001). Adding loneliness (block 3) explained an additional 9.6% of variance in personal recovery (*R*^2^ change = .096, β = −.34, *p* < .001; see Tables 2 and 3). Analysis in the schizophrenia subgroup showed similar results for loneliness predicting personal recovery (*n* = 121, *R*^2^ change = .050, β = −.25, *p* = .009; see Supplemental Material STs5 and 6).

**Table 2.** Model Summary for Loneliness Score Predicting Total Personal Recovery (QPR; *n* = 196).

Block	*F*	*R* ^2^	*R*^2^ change	Sig. *F* change
1^a^	1.63	.033	.033	0.17
2^b^	4.44	.124	.126	<0.001
3^c^	7.076	.219	.096	<0.001*
4^d^	6.37	.220	.001	0.63

a Predictors added to the model: age, sex, education level, country of birth.

b Predictors added to the model: hazardous alcohol use, cannabis use, nicotine use, total symptom severity (BPRS).

c Predictors added to the model: loneliness score.

d Interaction model: loneliness and symptom severity entered as centered variables in the block before the interaction term loneliness × symptom severity.

* Significant after Bonferroni correction (*p* = .05 / 3 = .017 > .001).

**Table 3.** Multiple Hierarchical Regression Results for Loneliness, With Personal Recovery (QPR) as Dependent Variable (*n* = 196).

Model	Variable	*B*	Std. error	Beta	*t*	Sig.
1	Constant	83.74	4.55		18.40	<.001
	Sex	−0.52	1.60	−.024	−0.33	0.74
	Age	−0.067	0.079	−.061	−0.85	0.40
	Education level	0.11	1.065	.008	0.11	0.92
	Birth country	3.82	1.55	.18	2.46	0.015
2	Constant	93.69	5.64		16.61	<.001
	Sex	0.089	1.57	.004	0.056	0.96
	Age	−0.039	0.076	−.035	−0.51	0.61
	Education level	−0.335	1.033	−.023	−0.32	0.75
	Birth country	3.114	1.51	.15	2.062	0.041
	Nicotine use	−2.13	1.63	−.10	−1.31	0.19
	Cannabis use	4.23	1.90	.17	2.23	0.027
	Hazardous alcohol use	4.43	1.87	.17	2.37	0.019
	Symptom severity	−0.32	0.073	−.31	−4.39	<.001
3	Constant	94.68	5.33		17.77	<.001
	Sex	−0.50	1.49	−.023	−0.34	0.74
	Age	−0.021	0.072	−.019	−0.30	0.77
	Education level	−0.75	0.98	−.051	−0.76	0.45
	Birth country	3.014	1.43	.14	2.11	0.036
	Nicotine use	−2.62	1.54	−.13	−1.71	0.090
	Cannabis use	4.47	1.79	.17	2.49	0.014
	Hazardous alcohol use	3.29	1.79	.13	1.85	0.066
	Symptom severity	−0.18	0.074	−.17	−2.44	0.016
	Loneliness	−1.95	0.40	−.34	−4.88	<.001
4	Constant	83.019	4.59		18.099	<.001
	Sex	−0.51	1.50	−.023	−0.34	0.73
	Age	−0.023	0.072	−.020	−0.31	0.75
	Education level	−0.73	0.98	−.049	−0.74	0.46
	Birth country	3.041	1.43	.14	2.13	0.035
	Nicotine use	−2.62	1.54	−.12	−1.70	0.091
	Cannabis use	4.52	1.80	.18	2.51	0.013
	Hazardous alcohol use	3.18	1.80	.12	1.77	0.079
	Symptom severity centered	−0.17	0.076	−.17	−2.25	0.026
	Loneliness centered	−1.94	0.40	−.34	−4.84	<.001
	Interaction Loneliness × Symptom severity	−0.017	0.036	−.032	−0.48	0.63

*Note*. The result for the interaction symptom severity × loneliness is displayed in model 4. Model = The regression model number (1–4); *B* = Unstandardized regression coefficient; *SE* = Standard error of the regression coefficient; β = Standardized regression coefficient; *t* = *t*-statistic. *p*-value < .05 indicates a statistically significant result.

No interaction effect (block 4) was observed between total symptom severity and loneliness on personal recovery (*n* = 196, *R*^2^ change = .001, β = −.032, *p* = .63). An exploratory analysis indicated no significant interaction effects between the four different symptom subscales and loneliness on personal recovery (see Supplemental Material STs7 and 8).

#### Subjective Discrimination

Visual inspection showed that subjective discrimination was non-normally distributed (*n* = 202), characterized by a high level of minimum scores resulting in a right-skewed distribution. One hundred ninety-six participants were included in the model, since six were excluded due to missing variables pairs. Patients most often experienced discrimination because of their psychiatric disorder (Supplemental Material ST9). Log, square root, cube root and inverse transformations did not lead to a normal distribution. Despite non-normality, discrimination was added to the model in block 3, considering the large sample size and robustness of regression methods while alternative models were deemed less suitable for the research question. Given that discrimination did not explain additional variance in personal recovery (*R*^2^ change = .00, β = .003, *p* = .77), only a model summary is shown in the main text (Table 4), full regression results are found in Supplemental Material ST10. No significant interaction effect between symptom severity and discrimination was observed on personal recovery. Analysis in the schizophrenia subsample (*n* = 121) showed similar non-significant results for discrimination predicting personal recovery and no significant interaction effect (Supplemental Material STs11 and 12).

**Table 4.** Model Summary for Subjective Discrimination Predicting Total Personal Recovery (QPR; *n* = 196).

Block	*F*	*R* ^2^	*R*^2^ change	Sig. *F* change
1^a^	1.63	.033	.033	0.17
2^b^	4.44	.160	.126	<0.001
3^c^	3.93	.160	.000	0.77
4^d^	3.54	.161	.001	0.56

a Predictors added to the model: age, sex, education level, country of birth.

b Predictors added to the model: hazardous alcohol use, cannabis use, nicotine use, total symptom severity (BPRS).

c Predictors added to the model: discrimination score.

d Interaction model: discrimination and symptom severity entered as centered variables in the block before the interaction term discrimination × symptom severity (*n* = 195 due to 1 missing interaction term).

#### Subjective Unsafety

Of 203 participants, 65 (31.9%) patients reported feeling unsafe in their neighborhood occasionally. It explained 2.8% additional variance in personal recovery after adjusting for covariates (*n* = 196; 7 participants were excluded pairwise due to missing variable pairs, *R*^2^ change = .028, β = −.17, *p* = .012), remaining significant after Bonferroni multiple testing correction. There was no significant interaction effect between feeling unsafe and symptom severity on personal recovery (Table 5 shows the model summary, full regression results are found in Supplemental Material ST13). Analysis in the schizophrenia subsample (*n* = 121) showed that feeling unsafe did not significantly explain variance in personal recovery (*R*^2^ change = .013, β = −.12, *p* = .19), with no significant interaction effect (Supplemental Material STs14 and 15).

**Table 5.** Model Summary for Occasionally Experiencing Neighborhood Unsafety Predicting Total Personal Recovery (QPR; *n* = 196).

Block	*F*	*R* ^2^	*R*^2^ change	Sig. *F* change
1^a^	1.50	.033	.033	0.17
2^b^	4.082	.160	.126	<0.001
3^c^	4.26	.187	.028	0.012*
4^d^	4.29	.188	.001	0.65

a Predictors added to the model: age, sex, education level, country of birth.

b Predictors added to the model: hazardous alcohol use, cannabis use, nicotine use, total symptom severity (BPRS).

c Predictors added to the model: neighborhood unsafety.

d Interaction model: symptom severity entered as centered variable in the block before the interaction term neighborhood unsafety × symptom severity.

* Significant after Bonferroni correction (*p* = .05 / 3 = .017 > .012).

A post-hoc model with subjective unsafety severity was performed within the subgroup (*n* = 65) reporting unsafety. The variable exhibited a non-normal distribution, for which logarithmic, square root, inverse and cube root transformations did not lead to a normal distribution. Despite this, unsafety severity was added to the model considering regression robustness, although the small sample size might have limited precision. No significant contribution to personal recovery was found (*n* = 56; 9 participants lacked data on unsafety severity score or missed data on variable pairs), *R*^2^ change = .009, β = −.011, *p* = .41). No significant interaction between unsafety severity and symptom severity was observed on personal recovery (see Supplemental Material STs16 and 17).

#### Victimization

Among 204 participants, 73 (35.8%) reported having been victim to any form of victimization in the past 12 months. Victimization did not explain additional variance in personal recovery (*n* = 196, *R*^2^ change = .000, β = −.001, *p* = .99; see Supplemental Material STs18 and 19). Analyses per crime category were unjustified given the small sample sizes per category (Supplemental Material ST20).

#### Exploratory Analyses

The level of loneliness significantly explained additional variance in both the intrapersonal scale (*n* = 196, *R*^2^ change = .103, β = −.35, *p* < .001) and the interpersonal scale (*n* = 188, *R*^2^ change = .043, β = −.23, *p* = .004; see Supplemental Material STs21–24), with the strongest effect on the former.

## Discussion

### Main Findings

The aim of the current study was to investigate the relationships between personal recovery and experiences of loneliness, discrimination, and neighborhood unsafety in community-treated patients with SSD. Loneliness explained 9.6% of additional variance in personal recovery after adjusting for multiple covariates, indicating that higher levels of loneliness are associated with lower levels of personal recovery. No significant interaction effect between symptom severity and loneliness was found on personal recovery, suggesting that the strength of the association does not vary by symptom severity. Subjective discrimination did not significantly explain additional variance in personal recovery. Subjective unsafety explained 2.8% of additional variance in personal recovery, indicating a small negative association, although severity of subjective unsafety did not contribute to the model. No significant interactions were found between symptom severity and discrimination or subjective unsafety on personal recovery.

### Comparison With Previous Literature and Interpretation of Current Findings

Current findings align with a study involving 159 patients with SSD, finding a correlation between lower loneliness and higher personal recovery levels (Roe et al., 2011). Similarly, a study in patients with severe mental illness reported that loneliness negatively predicted personal and clinical recovery (Wang et al., 2020). Additionally, a substantial body of literature has confirmed a positive relationship between social network size and social support and various recovery outcomes (Degnan et al., 2018; Norman et al., 2012; Song, 2017; Soundy et al., 2015) or quality of life (Ritsner et al., 2006) in SSD patients. Current findings correspond with the relevance of connectedness for personal recovery highlighted in previous literature (Leamy et al., 2011). Additionally, they align with qualitative studies emphasizing the role of friendship for patients’ recovery (Huckle et al., 2021) and reporting that patients’ feelings of connectedness with others were closely related to their experiences of hope and life purpose (Barut et al., 2016). A review on loneliness in patients with psychosis reported that loneliness was not only associated with smaller social networks but also with increased internalized stigma (Lim et al., 2018), with a substantial body of literature highlighting its negative impact on recovery and well-being outcomes (Arboleya-Faedo et al., 2023; Penas et al., 2024; Sarraf et al., 2022; Yanos et al., 2008). This is in line with the negative association with both intrapersonal and interpersonal aspects of personal recovery observed in current study, suggesting that the association with loneliness is not solely attributable to conceptual overlap between loneliness and interpersonal aspects. Although the direction of the association cannot be determined due to the cross-sectional nature of the current study, it could be hypothesized that loneliness affects both connectedness through social isolation as meaningfulness and hope through a reduced sense of belonging or self-stigma. This perspective may align with the conceptual distinction between social (lack of social contacts) and emotional (lack of intimate relationships) loneliness (Liu & Rook, 2013; Weiss, 1974). However, it should be stressed that the found association might also indicate bidirectionality in the relationship, with loneliness contributing to lower personal recovery and lower personal recovery contributing to higher loneliness.

The association between higher loneliness and lower personal recovery scores persisted after adjusting for total symptom severity (using the BPRS-E), and in the final model loneliness was a stronger predictor of personal recovery than total symptom severity. Additionally, the absence of a significant interaction effect between symptom severity and loneliness on personal recovery suggests that the association is not influenced by overall symptom severity.

No significant association between subjective discrimination and personal recovery was observed. These findings contrast with prior research suggesting a negative impact of discrimination on various recovery outcomes. One study in 90 patients with a psychotic disorder found that perceived discrimination was associated with poorer clinical and personal recovery (Mak et al., 2017). Another study in patients with first-episode psychosis reported negative associations between discrimination and various personal recovery subdomains (Laxmi et al., 2023). A review reported an association between higher positive symptom severity and higher perceived discrimination levels (Pearce et al., 2019), while in patients with severe mental illness an association was observed between discrimination and decreased access to social networks (Webber et al., 2014). In non-clinical samples, higher discrimination scores were consistently associated to depressive symptoms and worse self-rated health (Hudson et al., 2016; Schulz et al., 2006). The EDS was developed to measure discrimination in ethnic minority populations and not in psychiatric patients, while the nature of reported discrimination in the current study was more frequently due to psychiatric disorder than to ethnicity (see Supplemental Material ST9 for reported reasons). The use of the EDS to measure discrimination therefore may have resulted in not detecting an association with personal recovery, while an instrument measuring discrimination or stigma specifically developed for psychiatric patients might have been more sensitive, such as the discrimination and stigma scale (DISC; Thornicroft et al., 2009). Moreover, variability in the discrimination variable was limited, perhaps due to the use of a 4-point Likert scale, instead of a 6-point scale used in other studies (Hudson et al., 2016; Laxmi et al., 2023).

The small negative association between subjective unsafety and personal recovery, independent of symptom severity level, suggests that experiencing unsafety relates to lower personal recovery in community-dwelling patients with SSD, although unsafety severity did not explain additional variance in personal recovery. To the best of our knowledge, no previous study assessed the association between personal recovery and subjective unsafety. For victimization, reviews reported links with substance use, homelessness (Latalova et al., 2014; Maniglio, 2009) and higher illness severity (Khalifeh et al., 2016) in patients with severe mental illness, contrasting with current findings showing no association between victimization and personal recovery. Interestingly, in the current sample more participants reported feeling unsafe (33.2%) than in the general population of Amsterdam in 2019 (20.8%). In addition, the rate of traditional victimization (property or violent crime, or vandalism; 30.2%) was higher than in the general population (22.9%; Centraal Bureau voor de Statistiek, 2019). These comparisons may suggest that SSD patients experience unsafety more frequently than the general population, perhaps related to an increased vulnerability to violent victimization, as previously reported for patients with severe mental illness (Meijwaard et al., 2015).

### Implications for Treatment and Future Research

Current findings suggest that treatment focus for patients with SSD should extend beyond symptom remission, supported by prior research suggesting that personal recovery is complementary to clinical and functional recovery (Leendertse et al., 2021; Skar-Froding et al., 2021; Van Eck et al., 2018). They highlight the relevance of psychosocial interventions targeting loneliness in treating community-dwelling patients, regardless of illness severity. Helping patients expanding their social networks may reduce social isolation and increase their sense of belonging. Furthermore, positive psychological interventions might effectively reduce self-stigma (Lim et al., 2020; Pina et al., 2021). Healthcare and social services need to collaborate to provide helpful psychosocial interventions. Additionally, based on prior research highlighting the challenges of discrimination experiences for patients with SSD, and current observed association between feeling unsafe and lower personal recovery, healthcare and social workers should adopt awareness and an empathetic and open attitude toward patients susceptible to such experiences.

Future research should entail longitudinal investigations of associations between loneliness and various recovery outcomes to clarify causal pathways, and potential indirect pathways could be investigated. Investigating other interactional effects could help identify specific potential treatment targets. The effects of discrimination and unsafety on recovery warrants further investigation in diverse geographical contexts. Given the subjective nature of personal recovery, as well as loneliness, discrimination and unsafety, qualitative research assessing first-person perspectives may deepen understanding of their personal impacts.

### Strengths and Limitations

Strengths of current study were the representative sample of community-treated SSD patients and the large variety of covariates, allowing for robust adjustment of relationships. Furthermore, the use of self-report measures enabled investigation of subjective experiences of living environment and recovery, minimizing social desirability bias. One limitation was the cross-sectional design, precluding the assessment of causality in detected associations. Second, limited variability related to non-normal distributions in several outcome variables may have limited the ability to detect significant associations. Third, the EDS was used to measure discrimination experiences, despite its original development for ethnic minority groups. Fourth, we lacked specific data on ethnic descent, for which we used country of birth as a proxy. Fifth, generalizability may be limited since all patients were treated in the same capital with a high urbanicity level, while experiences of living environments differ across geographical contexts.

## Conclusion

Higher loneliness was associated with lower personal recovery in community-treated patients with SSD. Subjective unsafety was modestly negatively associated with personal recovery, while unsafety severity and discrimination did not contribute. Addressing loneliness deserves special attention from mental healthcare and social services, highlighting the need for close collaboration to enable effective psychosocial interventions. Further longitudinal and qualitative investigations could deepen understanding of personal recovery and its relation to negative environmental factors, potentially contributing to the development of more recovery-supportive psychosocial interventions.

## Supplemental Material

sj-docx-1-isp-10.1177_00207640261438835 – Supplemental material for The Relationship Between Personal Recovery and Experienced Loneliness, Discrimination or Neighborhood Unsafety in Patients With a Schizophrenia Spectrum Disorder Supplemental material, sj-docx-1-isp-10.1177_00207640261438835 for The Relationship Between Personal Recovery and Experienced Loneliness, Discrimination or Neighborhood Unsafety in Patients With a Schizophrenia Spectrum Disorder by Auke Jelsma, Marieke van der Pluijm, Menno Segeren, Astrid Vellinga, Henriëtte Heering, Annet van Bergen, Martijn Kikkert, Mariken de Koning and Lieuwe de Haan in International Journal of Social Psychiatry
