# Supplementary material for: The Relationship Between Personal Recovery and Experienced Loneliness, Discrimination or Neighborhood Unsafety in Patients With a Schizophrenia Spectrum Disorder
Source: Int J Soc Psychiatry. 2026 Apr 9;72(7):2100–11. doi: 10.1177/00207640261438835 (PMC13748483; doi:10.1177/00207640261438835)
Supplement: Supplementary file 1 [file sj-docx-1-isp-10.1177_00207640261438835.docx]

**Supplementary Material** forming part of:
The relationship between personal recovery and experienced loneliness, discrimination or neighborhood unsafety in patients with a schizophrenia spectrum disorder

**Supplementary Material ST1:** The Questionnaire of Personal Recovery (QPR) consisting of 22 items.

|  | Disagree strongly | Disagree | Neither agree nor disagree | Agree | Agree strongly |
| --- | --- | --- | --- | --- | --- |
| 1. I feel better about myself |  |  |  |  |  |
| 2. I feel able to take chances in life |  |  |  |  |  |
| 3. I am able to develop positive relationships with other people |  |  |  |  |  |
| 4. I feel part of society rather than isolated |  |  |  |  |  |
| 5. I am able to assert myself |  |  |  |  |  |
| 6. I feel that my life has a purpose |  |  |  |  |  |
| 7. My experiences have changed me for the better |  |  |  |  |  |
| 8. I have been able to come to terms with things that have happened |  |  |  |  |  |
| 9. I am basically strongly motivated to get better |  |  |  |  |  |
| 10. I can recognize the positive things I have done |  |  |  |  |  |
| 11. I am able to understand myself better |  |  |  |  |  |
| 12. I can take charge of my life |  |  |  |  |  |
| 13. I am able to access independent support |  |  |  |  |  |
| 14. I can weigh up the pros and cons of psychiatric treatment |  |  |  |  |  |
| 15. I feel my experiences have made me more sensitive towards  others |  |  |  |  |  |
| 16. Meeting people who have had similar experiences makes me feel  better |  |  |  |  |  |
| 17. My recovery has helped challenge other people’s views about getting better |  |  |  |  |  |
| 18. I am able to make sense of my distressing experiences |  |  |  |  |  |
| 19. I can actively engage with life |  |  |  |  |  |
| 20. I realize that the views of some mental health professionals is not the only way of looking at things |  |  |  |  |  |
| 21. I can take control of aspects of my life |  |  |  |  |  |
| 22. I can find the time to do the things I enjoy |  |  |  |  |  |

**Supplementary Material ST2**: The five questions which were used to assess severity of subjective neighborhood unsafety.

|  | Never or rarely | Sometimes | Often |
| --- | --- | --- | --- |
| How often do you not open the door at night due to not finding it safe? |  |  |  |
| How often do you take a detour or avoid certain places in your neighborhood to avoid places of unsafety? |  |  |  |
| How often do you feel unsafe when walking in your neighborhood at night? |  |  |  |
| How often do you feel unsafe when being alone at home at night? |  |  |  |
| How often are you afraid becoming victim of criminality? |  |  |  |

**Supplementary Material ST3:** Participants were asked whether they had been victim to any of the following 15 crimes:

1. House burglary

2. Theft from car

3. Car theft

4. Theft of other motor vehicle

5. Bicycle theft

6. Pickpocketing or robbery

7. Sexual crime

8. Threat

9. Violence

10. Other theft

11. Vandalism

12. Identity fraud

13. Sales fraud

14. Hacking

15. Cyberbullying

Example question for ‘’house burglary’’:

*Has your home ever been burglarized in the past 5 years, or has this been attempted?*

[if yes], *Has this also happened in the past twelve months?*

**Supplementary Material ST4**: Sensitivity analyses with an overview of imputed regression analyses per predictor (imputations were only done for missing covariates given the low missingness in predictors).

| Predictor | Pooled β coefficient | SE | t | Average R² change | p-value | N | Comparison with original analysis |
| --- | --- | --- | --- | --- | --- | --- | --- |
| Loneliness | -0.34 | 0.39 | -4.98 | 0.096 | <0.001 | 203 | Similar |
| Discrimination | 0.019 | 0.12 | 0.27 | 0.000 | 0.76 | 202 | Similar |
| Subjective unsafety (binary) | -0.17 | 1.53 | -2.51 | 0.027 | 0.013 | 203 | Similar |
| Subjective unsafety severity¹ | -0.11 | 0.53 | -0.83 | 0.0088 | 0.41 | 57 | Similar |
| Victimization | 0.0028 | 1.54 | 0.040 | 0.00 | 0.96 | 204 | Similar |
| Loneliness * Symptom severity | -0.030 | 0.035 | -0.46 | 0.001 | 0.65 | 201 | Similar |
| Discrimination * Symptom severity | -0.042 | 0.009 | -0.56 | 0.001 | 0.58 | 200 | Similar |
| Unsafety (binary) * Symptom severity | -0.0049 | 0.15 | -0.59 | 0.0014 | 0.56 | 202 | Similar |
| Unsafety severity * Symptom severity¹ | -0.069 | 0.048 | -0.38 | 0.0018 | 0.71 | 56 | Similar |
| Intrapersonal recovery | -0.35 | 0.33 | -5.29 | 0.10 | <0.001 | 203 | Similar |
| Interpersonal recovery | -0.23 | 0.11 | -2.93 | 0.043 | 0.0038 | 192 | Similar |

¹Within the group of patients feeling unsafe occasionally (n = 65).

**Supplementary Material ST5**: Model summary for loneliness predicting personal recovery (QPR) in the schizophrenia subgroup (N = 121). The result for the interaction total symptom severity * loneliness on personal recovery is displayed in the fourth model.

| Block | F | R² | R² change | Sig. F change |
| --- | --- | --- | --- | --- |
| 1¹ | 1.63 | 0.043 | 0.043 | 0.28 |
| 2² | 4.44 | 0.157 | 0.115 | 0.006 |
| 3³ | 6.98 | 0.208 | 0.050 | 0.009 |
| 4⁴ | 6.33 | 0.212 | 0.005 | 0.41 |

¹Predictors added to the model: age, sex, education level, country of birth
²Predictors added to the model: hazardous alcohol use, cannabis use, nicotine use, total symptom severity (BPRS)

³Predictors added to the model: loneliness

⁴Interaction model: interaction term centered loneliness * centered total symptoms

**Supplementary Material ST6:** Multiple hierarchical regression results in the schizophrenia subsample (n = 121) for loneliness, with personal recovery (QPR) as dependent variable and the interaction effect total symptom severity * loneliness displayed in the fourth model.

| Model |  | B | SE | Beta | t | p-value |
| --- | --- | --- | --- | --- | --- | --- |
| 1 | Constant | 87.23 | 5.66 |  | 15.42 | <0.001 |
|  | Sex | 0.75 | 2.25 | 0.031 | 0.33 | 0.74 |
|  | Age | -0.14 | 0.10 | -0.13 | -1.43 | 0.16 |
|  | Education level | -1.10 | 1.41 | -0.071 | -0.78 | 0.44 |
|  | Birth country | 3.14 | 1.98 | 0.15 | 1.59 | 0.12 |
| 2 | Constant | 91.96 | 7.56 |  | 12.18 | <0.001 |
|  | Sex | 1.21 | 2.23 | 0.051 | 0.54 | 0.59 |
|  | Age | -0.10 | 0.099 | -0.094 | -1.029 | 0.31 |
|  | Education level | -1.32 | 1.39 | -0.086 | -0.95 | 0.34 |
|  | Birth country | 2.53 | 1.97 | 0.12 | 1.29 | 0.20 |
|  | Nicotine use | -3.43 | 2.17 | -0.17 | -1.59 | 0.12 |
|  | Cannabis use | 6.97 | 2.25 | 0.30 | 3.094 | 0.002 |
|  | Hazardous alcohol use | 2.90 | 2.30 | 0.12 | 1.26 | 0.21 |
|  | Symptom severity | -0.20 | 0.094 | -0.19 | -2.14 | 0.035 |
| 3 | Constant | 92.078 | 7.36 |  | 12.51 | <0.001 |
|  | Sex | 0.47 | 2.19 | 0.020 | 0.22 | 0.83 |
|  | Age | -0.073 | 0.097 | -0.067 | -0.75 | 0.46 |
|  | Education level | -1.61 | 1.36 | -0.10 | -1.18 | 0.24 |
|  | Birth country | 2.83 | 1.92 | 0.14 | 1.45 | 0.14 |
|  | Nicotine use | -3.45 | 2.11 | -0.17 | -1.64 | 0.10 |
|  | Cannabis use | 6.54 | 2.20 | 0.28 | 2.97 | 0.004 |
|  | Hazardous alcohol use | 2.48 | 2.25 | 0.10 | 1.10 | 0.27 |
|  | Symptom severity | -0.097 | 0.10 | -0.093 | -0.98 | 0.33 |
|  | Loneliness | -1.45 | 0.55 | -0.25 | -2.65 | 0.009 |
| 4 | Constant | 84.62 | 6.19 |  | 13.67 | <0.001 |
|  | Sex | 0.58 | 2.19 | 0.024 | 0.27 | 0.79 |
|  | Age | -0.076 | 0.097 | -0.070 | -0.78 | 0.44 |
|  | Education level | -1.67 | 1.36 | -0.11 | -1.22 | 0.22 |
|  | Birth country | 2.58 | 1.95 | 0.12 | 1.32 | 0.19 |
|  | Nicotine use | -3.67 | 2.13 | -0.18 | -1.72 | 0.088 |
|  | Cannabis use | 6.62 | 2.20 | 0.29 | 3.001 | 0.003 |
|  | Hazardous alcohol use | 2.62 | 2.26 | 0.11 | 1.16 | 0.25 |
|  | Symptom severity centered | -0.12 | 0.10 | -0.12 | -1.16 | 0.25 |
|  | Loneliness centered | -1.41 | 0.55 | -0.24 | -2.54 | 0.012 |
|  | Interaction Loneliness * total symptoms | 0.046 | 0.055 | .074 | 0.83 | 0.41 |

Model: Indicates the regression model number (1–4).

B: Unstandardized regression coefficient.

SE: Standard error of the regression coefficient.

β: Standardized regression coefficient.

t: t-statistic.

p-value < 0.05 indicates a statistically significant result

**Supplementary Material ST7**: Model summary for loneliness predicting personal recovery (QPR) (n = 195), with the four symptoms subscales entered as centered variables in the second model, and the interaction effects of the four symptom subscales * loneliness on personal recovery displayed in the fourth model.

| Block | F | R² | R² change | Sig. F change |
| --- | --- | --- | --- | --- |
| 1¹ | 2.11 | 0.073 | 0.073 | 0.045 |
| 2² | 5.80 | 0.258 | 0.185 | <0.001 |
| 3³ | 7.055 | 0.317 | 0.059 | <0.001 |
| 4⁴ | 5.38 | 0.326 | 0.008 | 0.70 |

¹Predictors added to the model: age, sex, education level, country of birth, nicotine use, cannabis use, hazardous alcohol use
²Predictors added to the model: the four symptom subscales (centered)

³Predictors added to the model: loneliness (centered)

⁴Interaction model: interaction terms centered loneliness * centeral symptom subscale variables

**Supplementary Material ST8:** Multiple hierarchical regression results (n = 195) for loneliness predicting personal recovery (QPR), with the four symptoms subscales entered as centered variables in the second model, and the interaction effects of the four symptom subscales * loneliness on personal recovery displayed in the fourth model.

| Model |  | B | SE | Beta | t | p-value | VIF |
| --- | --- | --- | --- | --- | --- | --- | --- |
| 1 | Constant | 80.90 | 5.070 |  | 15.96 | <0.001 |  |
|  | Sex | 0.062 | 1.65 | 0.003 | .037 | 0.97 | 1.13 |
|  | Age | -0.035 | 0.080 | -0.032 | -0.45 | 0.66 | 1.048 |
|  | Education level | 0.23 | 1.076 | 0.016 | 0.22 | 0.83 | 1.069 |
|  | Birth country | 4.18 | 1.57 | 0.20 | 2.67 | 0.008 | 1.097 |
|  | Nicotine use | -2.74 | 1.70 | -0.13 | -1.61 | 0.11 | 1.32 |
|  | Cannabis use | 4.096 | 1.99 | 0.16 | 2.055 | 0.041 | 1.22 |
|  | Hazardous alcohol use | 3.90 | 1.96 | 0.15 | 1.99 | 0.048 | 1.16 |
| 2 | Constant | 79.50 | 4.64 |  | 17.15 | <0.001 |  |
|  | Sex | 0.68 | 1.51 | 0.031 | .45 | 0.65 | 1.15 |
|  | Age | -8.017 | 0.072 | -0.015 | -.23 | 0.82 | 1.055 |
|  | Education level | 0.48 | 1.00 | 0.032 | .48 | 0.63 | 1.13 |
|  | Birth country | 2.38 | 1.45 | 0.11 | 1.65 | 0.10 | 1.15 |
|  | Nicotine use | -2.045 | 1.55 | -0.097 | -1.32 | 0.19 | 1.33 |
|  | Cannabis use | 2.88 | 1.86 | 0.11 | 1.55 | 0.12 | 1.29 |
|  | Hazardous alcohol use | 3.76 | 1.80 | 0.15 | 2.095 | 0.038 | 1.20 |
|  | Positive symptoms centered | 0.18 | 0.18 | 0.076 | 1.00 | 0.32 | 1.45 |
|  | Negative symptoms centered | -0.63 | 0.26 | -0.18 | -2.44 | 0.016 | 1.32 |
|  | Affective symptoms centered | -1.12 | 0.20 | -0.45 | -5.62 | <0.001 | 1.57 |
|  | Excitement/Disorganization symptoms centered | 0.44 | 0.25 | 0.14 | 1.73 | 0.085 | 1.64 |
| 3 | Constant | 80.86 | 4.47 |  | 18.077 | <0.001 |  |
|  | Sex | 0.068 | 1.46 | 0.003 | .046 | 0.96 | 1.16 |
|  | Age | -0.007 | 0.069 | -0.007 | -.11 | 0.92 | 1.057 |
|  | Education level | -0.032 | 0.97 | -0.002 | -.034 | 0.97 | 1.15 |
|  | Birth country | 2.47 | 1.39 | 0.12 | 1.78 | 0.077 | 1.15 |
|  | Nicotine use | -2.45 | 1.49 | -0.12 | -1.64 | 0.10 | 1.34 |
|  | Cannabis use | 3.29 | 1.79 | 0.13 | 1.84 | 0.068 | 1.30 |
|  | Hazardous alcohol use | 2.92 | 1.74 | 0.11 | 1.68 | 0.095 | 1.22 |
|  | Positive symptoms centered | 0.18 | 0.17 | 0.078 | 1.063 | 0.29 | 1.45 |
|  | Negative symptoms centered | -0.53 | 0.25 | -0.15 | -2.14 | 0.034 | 1.33 |
|  | Affective symptoms centered | -0.84 | 0.21 | -0.34 | -4.097 | <0.001 | 1.78 |
|  | Excitement/Disorganization symptoms centered | 0.44 | 0.24 | 0.14 | 1.82 | 0.070 | 1.64 |
|  | Loneliness centered | -1.59 | 0.40 | -0.28 | -3.97 | <0.001 | 1.29 |
| 4 | Constant | 81.46 | 4.52 |  | 18.017 | <0.001 |  |
|  | Sex | -0.14 | 1.47 | -0.006 | -0.097 | 0.92 | 1.17 |
|  | Age | -0.004 | 0.070 | -0.003 | -0.051 | 0.96 | 1.074 |
|  | Education level | -0.14 | 0.98 | -0.009 | -0.14 | 0.89 | 1.17 |
|  | Birth country | 2.43 | 1.40 | 0.12 | 1.74 | 0.084 | 1.15 |
|  | Nicotine use | -2.74 | 1.53 | -0.13 | -1.80 | 0.074 | 1.39 |
|  | Cannabis use | 3.38 | 1.82 | 0.13 | 1.86 | 0.065 | 1.33 |
|  | Hazardous alcohol use | 2.82 | 1.80 | 0.11 | 1.57 | 0.12 | 1.29 |
|  | Positive symptoms centered | 0.12 | 0.18 | 0.053 | 0.69 | 0.49 | 1.59 |
|  | Negative symptoms centered | -0.49 | 0.26 | -0.14 | -1.87 | 0.063 | 1.47 |
|  | Affective symptoms centered | -0.81 | 0.22 | -0.32 | -3.69 | <0.001 | 2.006 |
|  | Excitement/Disorganization symptoms centered | 0.52 | 0.26 | 0.17 | 2.007 | 0.046 | 1.84 |
|  | Loneliness centered | -1.66 | 0.41 | -0.29 | -4.072 | <0.001 | 1.33 |
|  | Interaction loneliness x positive symptoms | 0.077 | 0.087 | 0.069 | 0.89 | 0.38 | 1.59 |
|  | Interaction loneliness x negative symptoms | -0.054 | 0.15 | -0.026 | -0.35 | 0.73 | 1.45 |
|  | Interaction loneliness x affective symptoms | -0.037 | 0.10 | -0.029 | -0.35 | 0.73 | 1.84 |
|  | Interaction loneliness x excitement/disorganization symptoms | -0.11 | 0.13 | -0.071 | -0.87 | 0.39 | 1.77 |

Model: Indicates the regression model number (1–4).

B: Unstandardized regression coefficient.

SE: Standard error of the regression coefficient.

β: Standardized regression coefficient.

t: t-statistic.

p-value < 0.05 indicates a statistically significant result
VIF = Variance Inflation Factor

**Supplementary Material ST9:** Reasons for experienced discrimination.

| Reasons for experienced discrimination | *n* = 202 |
| --- | --- |
| Psychiatric disorder  Skin color  Ethnic descent  Age  Gender  Religion  Length/weight  Other physical aspect  Physical defect  Sexual orientation  Other reason | 59 (28.9%)  32 (15.7%)  24 (11.8%)  2 (1.0%)  2 (1.0%)  5 (2.5%)  4 (2.0%)  9 (4.4%)  3 (1.5%)  3 (1.5%)  39 (19.1%) |

**Supplementary Material ST10**: Multiple hierarchical regression results for subjective discrimination, with personal recovery (QPR) as dependent variable (N = 196). The interaction symptom severity * discrimination is displayed in the fourth model.

| Model |  | B | SE | Beta | t | p-value |
| --- | --- | --- | --- | --- | --- | --- |
| 1 | Constant | 83.61 | 4.50 |  | 18.58 | <0.001 |
|  | Sex | -0.41 | 1.56 | -0.019 | -0.26 | 0.79 |
|  | Age | -0.063 | 0.078 | -0.057 | -0.81 | 0.42 |
|  | Education level | 0.039 | 1.06 | 0.003 | 0.037 | 0.97 |
|  | Birth country | 3.56 | 1.51 | 0.17 | 2.36 | 0.019 |
| 2 | Constant | 93.47 | 5.62 |  | 16.63 | <0.001 |
|  | Sex | 0.14 | 1.54 | 0.006 | 0.091 | 0.93 |
|  | Age | -0.039 | 0.075 | -0.035 | -0.52 | 0.60 |
|  | Education level | -0.37 | 1.03 | -0.024 | -0.35 | 0.72 |
|  | Birth country | 2.93 | 1.48 | 0.14 | 1.98 | 0.050 |
|  | Nicotine use | -1.83 | 1.58 | -0.09 | -1.16 | 0.25 |
|  | Cannabis use | 3.75 | 1.87 | 0.15 | 2.01 | 0.046 |
|  | Hazardous alcohol use | 4.28 | 1.87 | 0.16 | 2.29 | 0.023 |
|  | Symptom severity | -0.31 | 0.072 | -0.295 | -4.32 | <0.001 |
| 3 | Constant | 93.52 | 5.81 |  | 16.10 | <0.001 |
|  | Sex | 0.14 | 1.54 | 0.006 | 0.092 | 0.93 |
|  | Age | -0.039 | 0.076 | -0.035 | -0.52 | 0.60 |
|  | Education level | -0.36 | 1.03 | -0.024 | -0.35 | 0.73 |
|  | Birth country | 2.93 | 1.49 | 0.14 | 1.97 | 0.050 |
|  | Nicotine use | -1.83 | 1.58 | -0.09 | -1.16 | 0.25 |
|  | Cannabis use | 3.75 | 1.88 | 0.15 | 2.00 | 0.047 |
|  | Hazardous alcohol use | 4.28 | 1.88 | 0.16 | 2.28 | 0.023 |
|  | Symptom severity | -0.31 | 0.075 | -0.294 | -4.13 | <0.001 |
|  | Discrimination | -0.004 | 0.12 | -0.003 | -0.037 | 0.97 |
| 4 | Constant | 81.80 | 5.07 |  | 16.15 | <0.001 |
|  | Sex | 0.22 | 1.57 | 0.010 | 0.14 | 0.89 |
|  | Age | -0.039 | 0.076 | -0.035 | -0.51 | 0.61 |
|  | Education level | -0.36 | 1.04 | -0.024 | -0.34 | 0.73 |
|  | Birth country | 2.97 | 1.50 | 0.14 | 1.98 | 0.049 |
|  | Nicotine use | -1.85 | 1.59 | -0.088 | -1.16 | 0.25 |
|  | Cannabis use | 3.76 | 1.89 | 0.15 | 2.00 | 0.047 |
|  | Hazardous alcohol use | 4.25 | 1.89 | 0.16 | 2.26 | 0.025 |
|  | Symptom severity centered | -0.27 | 0.14 | -0.26 | -1.96 | 0.052 |
|  | Discrimination centered | 0.005 | 0.12 | 0.003 | 0.042 | 0.97 |
|  | Interaction Discrimination * Symptom severity | -0.003 | 0.009 | -0.046 | -0.35 | 0.73 |

Model: Indicates the regression model number (1–4).

B: Unstandardized regression coefficient.

SE: Standard error of the regression coefficient.

β: Standardized regression coefficient.

t: t-statistic.

p-value < 0.05 indicates a statistically significant result.

**Supplementary Material ST11** Model summary for subjective discrimination predicting total personal recovery (QPR) in the schizophrenia sample (n = 121).

| Block | F | R² | R² change | Sig. F change |
| --- | --- | --- | --- | --- |
| 1¹ | 1.29 | 0.043 | 0.043 | 0.28 |
| 2² | 2.61 | 0.097 | 0.115 | 0.006 |
| 3³ | 2.31 | 0.090 | 0.001 | 0.80 |
| 4⁴ | 2.063 | 0.082 | 0.001 | 0.68 |

¹Predictors added to the model: age, sex, education level, country of birth
²Predictors added to the model: hazardous alcohol use, cannabis use, nicotine use, total symptom severity (BPRS)

³Predictors added to the model: discrimination score

⁴Interaction model: discrimination and symptom severity entered as centered variables in the block before the interaction term discrimination * symptom severity (N = 120)

**Supplementary Material ST12**: Multiple hierarchical regression results for subjective discrimination, with personal recovery (QPR) as dependent variable in the schizophrenia subsample (N = 121). The interaction symptom severity * discrimination is displayed in the fourth model.

| Model |  | B | SE | Beta | t | p-value |
| --- | --- | --- | --- | --- | --- | --- |
| 1 | Constant | 83.61 | 4.50 |  | 18.58 | <0.001 |
|  | Sex | -0.41 | 1.56 | -0.019 | -0.26 | 0.79 |
|  | Age | -0.063 | 0.078 | -0.057 | -0.81 | 0.42 |
|  | Education level | 0.039 | 1.06 | 0.003 | 0.037 | 0.97 |
|  | Birth country | 3.56 | 1.51 | 0.17 | 2.36 | 0.019 |
| 2 | Constant | 93.47 | 5.62 |  | 16.63 | <0.001 |
|  | Sex | 0.14 | 1.54 | 0.006 | 0.091 | 0.93 |
|  | Age | -0.039 | 0.075 | -0.035 | -0.52 | 0.60 |
|  | Education level | -0.37 | 1.03 | -0.024 | -0.35 | 0.72 |
|  | Birth country | 2.93 | 1.48 | 0.14 | 1.98 | 0.050 |
|  | Nicotine use | -1.83 | 1.58 | -0.09 | -1.16 | 0.25 |
|  | Cannabis use | 3.75 | 1.87 | 0.15 | 2.01 | 0.046 |
|  | Hazardous alcohol use | 4.28 | 1.87 | 0.16 | 2.29 | 0.023 |
|  | Symptom severity | -0.31 | 0.072 | -0.30 | -4.32 | <0.001 |
| 3 | Constant | 93.52 | 5.81 |  | 16.10 | <0.001 |
|  | Sex | 0.14 | 1.54 | 0.006 | 0.092 | 0.93 |
|  | Age | -0.039 | 0.076 | -0.035 | -0.52 | 0.60 |
|  | Education level | -0.36 | 1.03 | -0.024 | -0.35 | 0.73 |
|  | Birth country | 2.93 | 1.49 | 0.14 | 1.97 | 0.050 |
|  | Nicotine use | -1.83 | 1.58 | -0.09 | -1.16 | 0.25 |
|  | Cannabis use | 3.75 | 1.88 | 0.15 | 2.00 | 0.047 |
|  | Hazardous alcohol use | 4.28 | 1.88 | 0.16 | 2.28 | 0.023 |
|  | Symptom severity | -0.31 | 0.075 | -0.29 | -4.13 | <0.001 |
|  | Discrimination | -0.004 | 0.12 | -0.003 | -0.037 | 0.97 |
| 4 | Constant | 84.28 | 6.46 |  | 13.055 | <0.001 |
|  | Sex | 1.33 | 2.27 | 0.056 | 0.59 | 0.56 |
|  | Age | -0.099 | 0.10 | -0.091 | -0.98 | 0.33 |
|  | Education level | -1.36 | 1.42 | -0.088 | -0.96 | 0.34 |
|  | Birth country | 2.70 | 2.052 | 0.13 | 1.32 | 0.19 |
|  | Nicotine use | -3.36 | 2.21 | -0.16 | -1.52 | 0.13 |
|  | Cannabis use | 6.83 | 2.30 | 0.30 | 2.98 | 0.004 |
|  | Hazardous alcohol use | 2.79 | 2.35 | 0.12 | 1.19 | 0.24 |
|  | Symptom severity centered | -0.19 | 0.10 | -0.19 | -1.87 | 0.064 |
|  | Discrimination centered | 0.054 | 0.16 | 0.033 | 0.34 | 0.73 |
|  | Interaction Discrimination * Symptom severity | -0.005 | 0.012 | -0.040 | -0.41 | 0.68 |

Model: Indicates the regression model number (1–4).

B: Unstandardized regression coefficient.

SE: Standard error of the regression coefficient.

β: Standardized regression coefficient.

t: t-statistic.

p-value < 0.05 indicates a statistically significant result.

**Supplementary Material ST13**: Multiple hierarchical regression results for reporting feeling unsafe occasionally in the neighborhood, with personal recovery (QPR) as dependent variable (n = 196). The interaction symptom severity * sometimes feeling unsafe is displayed in the fourth model.

| Model | Variable | B | Std. Error | Beta | t | Sig. |
| --- | --- | --- | --- | --- | --- | --- |
| 1 | Constant | 83.74 | 4.55 |  | 18.40 | <0.001 |
|  | Sex | -0.52 | 1.60 | -0.024 | -0.33 | 0.74 |
|  | Age | -0.067 | 0.079 | -0.061 | -0.85 | 0.40 |
|  | Education level | 0.11 | 1.07 | 0.008 | 0.11 | 0.92 |
|  | Birth country | 3.82 | 1.55 | 0.18 | 2.46 | 0.015 |
| 2 | Constant | 93.69 | 5.64 |  | 16.61 | <0.001 |
|  | Sex | 0.089 | 1.57 | 0.004 | 0.056 | 0.96 |
|  | Age | -0.039 | 0.076 | -0.035 | -0.51 | 0.61 |
|  | Education level | -0.34 | 1.03 | -0.023 | -0.32 | 0.75 |
|  | Birth country | 3.11 | 1.51 | 0.15 | 2.06 | 0.041 |
|  | Nicotine use | -2.13 | 1.63 | -0.10 | -1.31 | 0.19 |
|  | Cannabis use | 4.23 | 1.90 | 0.17 | 2.23 | 0.027 |
|  | Hazardous alcohol use | 4.43 | 1.87 | 0.17 | 2.37 | 0.019 |
|  | Symptom severity | -0.32 | 0.073 | -0.31 | -4.39 | <0.001 |
| 3 | Constant | 92.91 | 5.57 |  | 16.68 | <0.001 |
|  | Sex | 0.41 | 1.56 | 0.018 | 0.26 | 0.79 |
|  | Age | -0.011 | 0.076 | -0.010 | -0.14 | 0.89 |
|  | Education level | -0.31 | 1.02 | -0.021 | -0.31 | 0.76 |
|  | Birth country | 2.41 | 1.52 | 0.11 | 1.59 | 0.11 |
|  | Nicotine use | -2.54 | 1.61 | -0.12 | -1.57 | 0.12 |
|  | Cannabis use | 4.28 | 1.87 | 0.17 | 2.29 | 0.023 |
|  | Hazardous alcohol use | 4.61 | 1.84 | 0.18 | 2.50 | 0.013 |
|  | Symptom severity | -0.30 | 0.072 | -0.29 | -4.17 | <0.001 |
|  | Feeling sometimes unsafe | -3.92 | 1.55 | -0.17 | -2.53 | 0.012 |
| 4 | Constant | 81.80 | 4.78 |  | 17.12 | <0.001 |
|  | Sex | 0.43 | 1.56 | 0.019 | 0.27 | 0.79 |
|  | Age | -0.012 | 0.076 | -0.011 | -0.16 | 0.87 |
|  | Education level | -0.30 | 1.02 | -0.021 | -0.30 | 0.77 |
|  | Birth country | 2.42 | 1.52 | 0.11 | 1.60 | 0.11 |
|  | Nicotine use | -2.45 | 1.63 | -0.11 | -1.50 | 0.13 |
|  | Cannabis use | 4.15 | 1.90 | 0.16 | 2.19 | 0.030 |
|  | Hazardous alcohol use | 4.45 | 1.88 | 0.17 | 2.37 | 0.019 |
|  | Symptom severity centered | -0.28 | 0.089 | -0.26 | -3.08 | 0.002 |
|  | Feelings sometimes unsafe centered | -3.85 | 1.56 | -0.17 | -2.47 | 0.014 |
|  | Interaction Feeling sometimes unsafe * Symptom severity | -0.070 | 0.15 | -0.039 | -0.46 | 0.65 |

Model: Indicates the regression model number (1–4).

B: Unstandardized regression coefficient.

SE: Standard error of the regression coefficient.

β: Standardized regression coefficient.

t: t-statistic.

p-value < 0.05 indicates a statistically significant result.

**Supplementary Material ST14** Model summary for occasionally experiencing neighborhood unsafety predicting total personal recovery (QPR) in the schizophrenia subsample (n = 121).

| Block | F | R² | R² change | Sig. F change |
| --- | --- | --- | --- | --- |
| 1¹ | 1.29 | 0.043 | 0.043 | 0.28 |
| 2² | 2.61 | 0.157 | 0.115 | 0.006 |
| 3³ | 2.54 | 0.170 | 0.013 | 0.19 |
| 4⁴ | 4.29 | 0.188 | 0.019 | 0.13 |

¹Predictors added to the model: age, sex, education level, country of birth
²Predictors added to the model: hazardous alcohol use, cannabis use, nicotine use, total symptom severity (BPRS)

³Predictors added to the model: neighborhood unsafety
⁴Interaction model: symptom severity entered as centered variable in the block before the interaction term neighborhood unsafety * symptom severity

**Supplementary Material ST15**: Multiple hierarchical regression results for reporting feeling unsafe occasionally in the neighborhood, with personal recovery (QPR) as dependent variable in the schizophrenia subsample (n = 121). The interaction symptom severity * sometimes feeling unsafe is displayed in the fourth model.

| Model | Variable | B | Std. Error | Beta | t | Sig. |
| --- | --- | --- | --- | --- | --- | --- |
| 1 | Constant | 87.23 | 5.66 |  | 15.42 | <0.001 |
|  | Sex | 0.75 | 2.25 | 0.031 | 0.33 | 0.74 |
|  | Age | -0.14 | 0.10 | -0.13 | -1.43 | 0.16 |
|  | Education level | -1.10 | 1.41 | -0.071 | -0.78 | 0.44 |
|  | Birth country | 3.14 | 1.98 | 0.15 | 1.59 | 0.12 |
| 2 | Constant | 91.96 | 7.56 |  | 12.17 | <0.001 |
|  | Sex | 1.21 | 2.23 | 0.051 | 0.54 | 0.59 |
|  | Age | -0.10 | 0.099 | -0.094 | -1.029 | 0.31 |
|  | Education level | -1.32 | 1.39 | -0.086 | -0.95 | 0.34 |
|  | Birth country | 2.53 | 1.97 | 0.12 | 1.29 | 0.20 |
|  | Nicotine use | -3.43 | 2.17 | -0.17 | -1.59 | 0.12 |
|  | Cannabis use | 6.97 | 2.25 | 0.30 | 3.094 | 0.002 |
|  | Hazardous alcohol use | 2.90 | 2.30 | 0.12 | 1.26 | 0.21 |
|  | Symptom severity | -0.20 | 0.094 | -0.19 | -2.14 | 0.035 |
| 3 | Constant | 90.26 | 7.64 |  | 11.82 | <0.001 |
|  | Sex | 1.68 | 2.25 | 0.071 | 0.75 | 0.46 |
|  | Age | -0.077 | 0.10 | -0.071 | -0.77 | 0.44 |
|  | Education level | -1.18 | 1.39 | -0.076 | -0.85 | 0.40 |
|  | Birth country | 2.090 | 1.99 | 0.10 | 1.050 | 0.30 |
|  | Nicotine use | -3.41 | 2.16 | -0.17 | -1.58 | 0.12 |
|  | Cannabis use | 6.79 | 2.25 | 0.29 | 3.021 | 0.003 |
|  | Hazardous alcohol use | 3.23 | 2.31 | 0.14 | 1.40 | 0.17 |
|  | Symptom severity | -0.18 | 0.095 | -0.17 | -1.89 | 0.061 |
|  | Feeling sometimes unsafe | -2.68 | 2.017 | -0.12 | -1.33 | 0.19 |
| 4 | Constant | 84.23 | 6.34 |  | 13.29 | <0.001 |
|  | Sex | 1.72 | 2.23 | 0.072 | 0.77 | 0.44 |
|  | Age | -0.098 | 0.10 | -0.091 | -0.98 | 0.30 |
|  | Education level | -1.13 | 1.38 | -0.073 | -0.81 | 0.42 |
|  | Birth country | 2.59 | 2.006 | 0.13 | 1.29 | 0.20 |
|  | Nicotine use | -2.40 | 2.24 | -0.12 | -1.070 | 0.20 |
|  | Cannabis use | 6.14 | 2.27 | 0.27 | 2.70 | 0.008 |
|  | Hazardous alcohol use | 2.25 | 2.38 | 0.095 | 0.95 | 0.35 |
|  | Symptom severity centered | -0.047 | 0.13 | -0.045 | -0.37 | 0.72 |
|  | Feeling sometimes unsafe | -2.36 | 2.015 | -0.11 | -1.17 | 0.24 |
|  | Interaction Feeling sometimes unsafe * Symptom severity | -0.31 | 0.20 | -0.19 | -1.53 | 0.13 |

**Supplementary Material ST16**: Model summary for severity of subjective unsafety predicting total personal recovery (QPR) within the group reporting feeling unsafe occasionally (N = 56).

| Block | F | R² | R² change | Sig. F change |
| --- | --- | --- | --- | --- |
| 1¹ | 2.74 | 0.177 | 0.174 | 0.039 |
| 2² | 3.87 | 0.397 | 0.220 | 0.005 |
| 3³ | 3.49 | 0.406 | 0.009 | 0.41 |
| 4⁴ | 3.03 | 0.408 | 0.002 | 0.71 |

¹Predictors added to the model: age, sex, education level, country of birth
²Predictors added to the model: hazardous alcohol use, cannabis use, nicotine use, total symptom severity (BPRS)

³Predictors added to the model: neighborhood unsafety score

⁴Interaction model: symptom severity and subjective unsafety severity were entered as centered variables in the block before the interaction term subjective unsafety severity * symptom severity (N = 55 due to 1 missing interaction term)

**Supplementary Material ST17**: Multiple hierarchical regression results for neighborhood unsafety severity, with personal recovery (QPR) as dependent variable within the group reporting feeling unsafe sometimes (N = 56). The interaction symptom severity * unsafety severity is displayed in the fourth model (N = 55 due to 1 missing interaction term).

| Model | Variable | B | Std. Error | Beta | t | Sig. |
| --- | --- | --- | --- | --- | --- | --- |
| 1 | Constant | 101.67 | 8.17 |  | 12.44 | <0.001 |
|  | Sex | -4.11 | 2.61 | -0.204 | -1.58 | 0.12 |
|  | Age | -0.364 | 0.146 | -0.339 | -2.49 | 0.02 |
|  | Education level | -0.76 | 1.98 | -0.048 | -0.38 | 0.71 |
|  | Birth country | 5.26 | 2.92 | 0.243 | 1.80 | 0.08 |
| 2 | Constant | 114.08 | 9.75 |  | 11.70 | <0.001 |
|  | Sex | -2.79 | 2.41 | -0.138 | -1.16 | 0.25 |
|  | Age | -0.356 | 0.134 | -0.331 | -2.66 | 0.01 |
|  | Education level | -0.89 | 1.83 | -0.057 | -0.49 | 0.63 |
|  | Birth country | 5.25 | 2.68 | 0.243 | 1.96 | 0.06 |
|  | Nicotine use | 2.75 | 2.83 | 0.139 | 0.97 | 0.34 |
|  | Cannabis use | 1.43 | 3.23 | 0.057 | 0.44 | 0.66 |
|  | Hazardous alcohol use | 1.70 | 3.19 | 0.071 | 0.53 | 0.60 |
|  | Symptom severity | -0.423 | 0.117 | -0.443 | -3.61 | <.001 |
| 3 | Constant | 116.31 | 10.15 |  | 11.46 | <0.001 |
|  | Sex | -2.43 | 2.46 | -0.120 | -0.99 | 0.33 |
|  | Age | -0.365 | 0.135 | -0.340 | -2.71 | 0.01 |
|  | Education level | -1.11 | 1.85 | -0.071 | -0.60 | 0.55 |
|  | Birth country | 5.48 | 2.70 | 0.253 | 2.03 | 0.05 |
|  | Nicotine use | 2.38 | 2.87 | 0.120 | 0.83 | 0.41 |
|  | Cannabis use | 1.17 | 3.25 | 0.046 | 0.36 | 0.72 |
|  | Hazardous alcohol use | 2.43 | 3.32 | 0.102 | 0.73 | 0.47 |
|  | Symptom severity | -0.378 | 0.130 | -0.395 | -2.91 | 0.01 |
|  | Unsafety sum score | -0.44 | 0.54 | -0.111 | -0.83 | 0.41 |
| 4 | Constant | 98.71 | 8.70 |  | 11.35 | <0.001 |
|  | Sex | -2.38 | 2.51 | -0.118 | -0.95 | 0.35 |
|  | Age | -0.360 | 0.138 | -0.335 | -2.61 | 0.01 |
|  | Education level | -0.92 | 1.96 | -0.059 | -0.47 | 0.64 |
|  | Birth country | 5.50 | 2.76 | 0.254 | 1.99 | 0.05 |
|  | Nicotine use | 2.45 | 2.94 | 0.124 | 0.83 | 0.41 |
|  | Cannabis use | 1.35 | 3.36 | 0.053 | 0.40 | 0.69 |
|  | Hazardous alcohol use | 2.33 | 3.40 | 0.097 | 0.69 | 0.50 |
|  | Symptom severity centered | -0.329 | 0.187 | -0.344 | -1.76 | 0.09 |
|  | Discrimination centered | -0.42 | 0.55 | -0.105 | -0.76 | 0.45 |
|  | Interaction Unsafety sum score * Symptom severity | -0.018 | 0.049 | -0.069 | -0.37 | 0.71 |

Model: Indicates the regression model number (1–4).

B: Unstandardized regression coefficient.

SE: Standard error of the regression coefficient.

β: Standardized regression coefficient.

t: t-statistic.

p-value < 0.05 indicates a statistically significant result.

**Supplementary Material ST18**: Model summary for any type of victimization predicting total personal recovery (QPR) (N = 196).

| Block | F | R² | R² change | Sig. F change |
| --- | --- | --- | --- | --- |
| 1¹ | 1.63 | 0.033 | 0.033 | 0.17 |
| 2² | 4.44 | 0.160 | 0.126 | <0.001 |
| 3³ | 3.92 | 0.160 | 0.000 | 0.99 |

¹Predictors added to the model: age, sex, education level, country of birth
²Predictors added to the model: hazardous alcohol use, cannabis use, nicotine use, total symptom severity (BPRS)

³Any type of victimization in the past 12 months

**Supplementary Material ST19**: Multiple hierarchical regression results for any type of victimization predicting total personal recovery (QPR) (N = 196).

| Model | Variable | B | Std. Error | Beta | t | Sig. |
| --- | --- | --- | --- | --- | --- | --- |
| 1 | Constant | 83.74 | 4.55 |  | 18.40 | <.001 |
|  | Sex | -0.52 | 1.60 | -0.024 | -0.33 | 0.74 |
|  | Age | -0.067 | 0.079 | -0.061 | -0.85 | 0.40 |
|  | Education level | 0.112 | 1.07 | 0.008 | 0.11 | 0.92 |
|  | Birth country | 3.82 | 1.55 | 0.180 | 2.46 | 0.02 |
| 2 | Constant | 93.69 | 5.64 |  | 16.61 | <.001 |
|  | Sex | 0.089 | 1.57 | 0.004 | 0.06 | 0.96 |
|  | Age | -0.04 | 0.08 | -0.035 | -0.51 | 0.61 |
|  | Education level | -0.34 | 1.03 | -0.023 | -0.32 | 0.75 |
|  | Birth country | 3.11 | 1.51 | 0.147 | 2.06 | 0.04 |
|  | Nicotine use | -2.13 | 1.63 | -0.101 | -1.31 | 0.19 |
|  | Cannabis use | 4.23 | 1.90 | 0.165 | 2.23 | 0.03 |
|  | Hazardous alcohol use | 4.43 | 1.87 | 0.172 | 2.37 | 0.02 |
|  | Symptom severity | -0.318 | 0.073 | -0.305 | -4.39 | <.001 |
| 3 | Constant | 93.69 | 5.66 |  | 16.56 | <.001 |
|  | Sex | 0.088 | 1.58 | 0.004 | 0.06 | 0.96 |
|  | Age | -0.04 | 0.08 | -0.035 | -0.51 | 0.61 |
|  | Education level | -0.335 | 1.04 | -0.023 | -0.32 | 0.75 |
|  | Birth country | 3.11 | 1.52 | 0.147 | 2.05 | 0.04 |
|  | Nicotine use | -2.13 | 1.63 | -0.101 | -1.31 | 0.19 |
|  | Cannabis use | 4.23 | 1.91 | 0.165 | 2.22 | 0.03 |
|  | Hazardous alcohol use | 4.43 | 1.90 | 0.172 | 2.33 | 0.02 |
|  | Symptom severity | -0.318 | 0.074 | -0.305 | -4.27 | <.001 |
|  | Any type of victimization | -0.015 | 1.57 | -0.001 | -0.01 | 0.99 |

Model: Indicates the regression model number (1–4).

B: Unstandardized regression coefficient.

SE: Standard error of the regression coefficient.

β: Standardized regression coefficient.

t: t-statistic.

p-value < 0.05 indicates a statistically significant result.

**Supplementary Material ST20:** 15 different crimes categories were further specified in two broad crime categories (traditional and online crime) and five more specific crime categories (property crime, violent crime, vandalism, identity- and sales fraud and cybercrime- or bullying).

| Divided in two broad crime categories: Traditional crime (property, violent, vandalism)  Online crime (identity fraud, cybercrime)  Specified in five crime categories:  Property crime (1 -6, 10)  Violent crime (7-9)  Vandalism (11)  Identity and sales fraud (12, 13)  Cybercrime, -bullying (14, 15) | 58 (28.4%)  31 (15.2%)  38 (18.6%)  30 (14.7%)  9 (4.4%)  20 (9.8%)  16 (7.9%) |
| --- | --- |

**Supplementary Material ST21**: Exploratory analysis: model summary of the multiple hierarchical regression analysis with the intrapersonal subscale of personal recovery as dependent variable and loneliness as independent variable (N = 196).

| Block | F | R² | R² change | Sig. F change |
| --- | --- | --- | --- | --- |
| 1¹ | 1.67 | 0.034 | 0.030 | 0.16 |
| 2² | 5.39 | 0.166 | 0.154 | <0.001 |
| 3³ | 8.45 | 0.290 | 0.103 | <0.001 |

¹Predictors added to the model: age, sex, education level, country of birth
²Predictors added to the model: alcohol hazardous use, cannabis use, nicotine use, total symptom severity (BPRS)

³Predictors added to the model: loneliness

**Supplementary Material ST22**: Exploratory analysis: results of the multiple hierarchical regression analysis with the intrapersonal subscale of personal recovery as dependent variable and loneliness as independent variable (N = 196*).

| Model | Variable | B | Std. Error | Beta | t | Sig. |
| --- | --- | --- | --- | --- | --- | --- |
| 1 | Constant | 66.087 | 3.95 |  | 16.73 | <.001 |
|  | Sex | -0.076 | 1.39 | -0.004 | -0.055 | 0.96 |
|  | Age | -0.054 | 0.069 | -0.056 | -0.78 | 0.43 |
|  | Education level | -0.46 | 0.92 | -0.036 | -0.50 | 0.62 |
|  | Birth country | 3.18 | 1.35 | 0.17 | 2.36 | 0.019 |
| 2 | Constant | 77.54 | 4.81 |  | 16.11 | <.001 |
|  | Sex | 0.36 | 1.34 | 0.019 | 0.27 | 0.80 |
|  | Age | -0.035 | 0.065 | -0.037 | -0.54 | 0.59 |
|  | Education level | -1.03 | 0.88 | -0.08 | -1.17 | 0.24 |
|  | Birth country | 2.22 | 1.29 | 0.12 | 1.72 | 0.087 |
|  | Nicotine use | -2.11 | 1.39 | -0.12 | -1.52 | 0.13 |
|  | Cannabis use | 3.97 | 1.62 | 0.18 | 2.45 | 0.015 |
|  | Hazardous alcohol use | 2.81 | 1.59 | 0.13 | 1.76 | 0.080 |
|  | Symptom severity | -0.32 | 0.062 | -0.36 | -5.20 | <.001 |
| 3 | Constant | 78.44 | 4.52 |  | 17.37 | <.001 |
|  | Sex | -0.17 | 1.26 | -0.009 | -0.14 | 0.89 |
|  | Age | -0.019 | 0.061 | -0.020 | -0.32 | 0.75 |
|  | Education level | -1.40 | 0.83 | -0.11 | -1.69 | 0.092 |
|  | Birth country | 2.13 | 1.21 | 0.12 | 1.76 | 0.079 |
|  | Nicotine use | -2.56 | 1.30 | -0.14 | -1.96 | 0.051 |
|  | Cannabis use | 4.19 | 1.52 | 0.188 | 2.76 | 0.006 |
|  | Hazardous alcohol use | 1.78 | 1.51 | 0.080 | 1.18 | 0.24 |
|  | Symptom severity | -0.20 | 0.063 | -0.22 | -3.15 | 0.002 |
|  | Loneliness | -1.76 | 0.34 | -0.35 | -5.19 | <.001 |

Model: Indicates the regression model number (1–4).

B: Unstandardized regression coefficient.

SE: Standard error of the regression coefficient.

β: Standardized regression coefficient.

t: t-statistic.

p-value < 0.05 indicates a statistically significant result.

**Supplementary Material ST23**: Exploratory analysis: model summary of the multiple hierarchical regression analysis with the interpersonal subscale of personal recovery as dependent variable and loneliness as independent variable (N = 188*).

| Block | F | R² | R² change | Sig. F change |
| --- | --- | --- | --- | --- |
| 1¹ | 0.70 | 0.015 | 0.015 | 0.60 |
| 2² | 1.032 | 0.044 | 0.025 | 0.29 |
| 3³ | 1.89 | 0.087 | 0.043 | 0.004 |

¹Predictors added to the model: age, sex, education level, country of birth
²Predictors added to the model: alcohol hazardous use, cannabis use, nicotine use, total symptom severity (BPRS)

³Predictors added to the model: loneliness
* For the interpersonal scale, more participants were excluded compared to the intrapersonal scale because of a lower number of items for the former, which made it more likely for participants to fail to meet the required 80% of items per variable, resulting in more excluded participants based on missing variables in this subscale.

**Supplementary Material ST24**: Exploratory analysis: results of the multiple hierarchical regression analysis with the interpersonal subscale of personal recovery as dependent variable and loneliness as independent variable (N = 188*).

| Model | Variable | B | Std. Error | Beta | t | Sig. |
| --- | --- | --- | --- | --- | --- | --- |
| 1 | Constant | 17.68 | 1.16 |  | 15.30 | <0.001 |
|  | Sex | 0.275 | 0.41 | 0.051 | 0.68 | 0.50 |
|  | Age | -0.015 | 0.020 | -0.055 | -0.74 | 0.46 |
|  | Education level | 0.34 | 0.27 | 0.093 | 1.25 | 0.21 |
|  | Birth country | 0.23 | 0.39 | 0.044 | 0.59 | 0.56 |
| 2 | Constant | 16.84 | 1.51 |  | 11.12 | <0.001 |
|  | Sex | 0.345 | 0.42 | 0.063 | 0.82 | 0.42 |
|  | Age | -0.009 | 0.020 | -0.032 | -0.43 | 0.67 |
|  | Education level | 0.40 | 0.28 | 0.11 | 1.43 | 0.15 |
|  | Birth country | 0.38 | 0.41 | 0.073 | 0.94 | 0.35 |
|  | Nicotine use | -0.25 | 0.44 | -0.047 | -0.56 | 0.57 |
|  | Cannabis use | 0.164 | 0.51 | 0.026 | 0.32 | 0.75 |
|  | Hazardous alcohol use | 1.15 | 0.50 | 0.18 | 2.29 | 0.02 |
|  | Symptom severity | 0.004 | 0.019 | 0.017 | 0.22 | 0.83 |
| 3 | Constant | 17.00 | 1.48 |  | 11.46 | <0.001 |
|  | Sex | 0.25 | 0.42 | 0.045 | 0.60 | 0.55 |
|  | Age | -0.006 | 0.020 | -0.021 | -0.29 | 0.77 |
|  | Education level | 0.33 | 0.27 | 0.090 | 1.21 | 0.23 |
|  | Birth country | 0.37 | 0.40 | 0.07 | 0.92 | 0.36 |
|  | Nicotine use | -0.33 | 0.43 | -0.063 | -0.76 | 0.45 |
|  | Cannabis use | 0.20 | 0.50 | 0.032 | 0.41 | 0.68 |
|  | Hazardous alcohol use | 0.96 | 0.50 | 0.15 | 1.94 | 0.05 |
|  | Symptom severity | 0.027 | 0.021 | 0.11 | 1.32 | 0.19 |
|  | Loneliness | -0.32 | 0.11 | -0.23 | -2.91 | 0.00 |

Model: Indicates the regression model number (1–4).

B: Unstandardized regression coefficient.

SE: Standard error of the regression coefficient.

β: Standardized regression coefficient.

t: t-statistic.

p-value < 0.05 indicates a statistically significant result.
* For the interpersonal scale, more participants were excluded compared to the intrapersonal scale because of a lower number of items for the former, which made it more likely for participants to fail to meet the required 80% of items per variable, resulting in more excluded participants based on missing variables in this subscale.
